# Sputum smear negative pulmonary tuberculosis: sensitivity and specificity of diagnostic algorithm

**DOI:** 10.1186/1756-0500-4-475

**Published:** 2011-11-01

**Authors:** Hedwiga F Swai, Ferdinand M Mugusi, Jessie K Mbwambo

**Affiliations:** 1 Department of Internal Medicine Muhimbili National Hospital, Dar-es-salaam, +255 Tanzania; 2 Department of Internal Medicine Muhimbili University of Health and Allied Sciences. Dar-es-salaam, +255 Tanzania; 3 Department of Psychiatry Muhimbili National Hospital. Dar-es-salaam, +255 Tanzania

**Keywords:** Sputum smear negative, Human Immunodeficiency Virus, Symptoms

## Abstract

**Background:**

The diagnosis of pulmonary tuberculosis in patients with Human Immunodeficiency Virus (HIV) is complicated by the increased presence of sputum smear negative tuberculosis. Diagnosis of smear negative pulmonary tuberculosis is made by an algorithm recommended by the National Tuberculosis and Leprosy Programme that uses symptoms, signs and laboratory results.

The objective of this study is to determine the sensitivity and specificity of the tuberculosis treatment algorithm used for the diagnosis of sputum smear negative pulmonary tuberculosis.

**Methods:**

A cross-section study with prospective enrollment of patients was conducted in Dar-es-Salaam Tanzania. For patients with sputum smear negative, sputum was sent for culture. All consenting recruited patients were counseled and tested for HIV. Patients were evaluated using the National Tuberculosis and Leprosy Programme guidelines and those fulfilling the criteria of having active pulmonary tuberculosis were started on anti tuberculosis therapy. Remaining patients were provided appropriate therapy. A chest X-ray, mantoux test, and Full Blood Picture were done for each patient. The sensitivity and specificity of the recommended algorithm was calculated. Predictors of sputum culture positive were determined using multivariate analysis.

**Results:**

During the study, 467 subjects were enrolled. Of those, 318 (68.1%) were HIV positive, 127 (27.2%) had sputum culture positive for Mycobacteria Tuberculosis, of whom 66 (51.9%) were correctly treated with anti-Tuberculosis drugs and 61 (48.1%) were missed and did not get anti-Tuberculosis drugs. Of the 286 subjects with sputum culture negative, 107 (37.4%) were incorrectly treated with anti-Tuberculosis drugs. The diagnostic algorithm for smear negative pulmonary tuberculosis had a sensitivity and specificity of 38.1% and 74.5% respectively. The presence of a dry cough, a high respiratory rate, a low eosinophil count, a mixed type of anaemia and presence of a cavity were found to be predictive of smear negative but culture positive pulmonary tuberculosis.

**Conclusion:**

The current practices of establishing pulmonary tuberculosis diagnosis are not sensitive and specific enough to establish the diagnosis of Acid Fast Bacilli smear negative pulmonary tuberculosis and over treat people with no pulmonary tuberculosis.

## Background

There has been a sharp rise in the incidence of pulmonary tuberculosis (PTB) worldwide since the mid 1980's, particularly in the Sub-Saharan African region. This has been attributed mainly to the appearance and wide spread of Human Immunodeficiency Virus (HIV) infection on the continent [1-3]. For the diagnosis of PTB the detection of Acid Fast Bacilli (AFB) in expectorated sputum is still crucial, especially in developing countries of Sub-Saharan Africa, where other facilities including sputum culture for Mycobacterium Tuberculosis (MTB) are unavailable or are prohibitively expensive. When AFB is detected in sputum, the diagnosis of PTB is certain. However diagnostic problem start when patients with suspected PTB have a negative sputum smear [4]. It has always been recognized that a proportion of patients are sputum smear negative using the Ziehl-Nelseen (ZN) stain, the commonly used stain in most laboratories in the region to detect AFB in sputum. This is a simple, rapid and cheap test but lacks sensitivity of a single sputum test [4]. About 5000 bacilli per milliliter of sputum must be present for it to be positive. However it has been reported that multiple sputum tests in a good laboratory can give a sensitivity of about 90% [5]. Sputum smear using ZN stain for AFB seems to be even less sensitive in patients with HIV associated PTB. With the sharp rise of PTB in countries which are worst affected by the HIV epidemics, the number of patients with suspected PTB who are sputum smear negative has increased [5].

**Figure 1:**
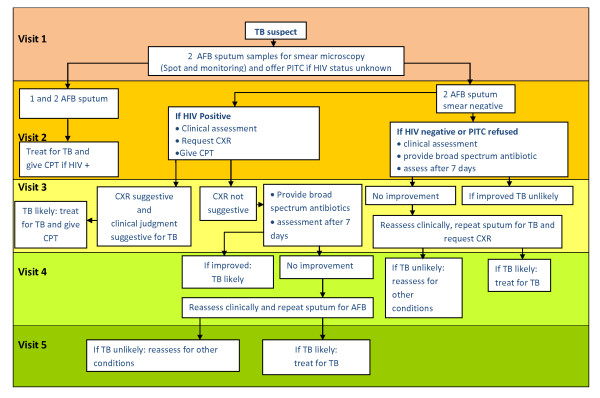
**Flowchart on the diagnosis of pulmonary TB in children above 6 years and adults**.

Chest radiography is not always helpful in smear negative patients. The radiographic distinction between active and inactive tuberculosis can be difficult and appearance may be atypical due to other infections in HIV positive patients [4]. In fact, substantial numbers of patients are treated for tuberculosis without definitive diagnostic criteria [5]. With the advent of HIV associated tuberculosis with more frequent smear negative tuberculosis, the role of culture in TB control programs may need to be reassessed [4]. In countries where resources are limited, and where the use of chest X-rays may be inadequate due to the cost as well as atypical presentation found in HIV infected patients, clinical and/or laboratory characteristics which are able to identify smear negative but culture positive PTB are required. The Tanzania National TB and Leprosy Programme uses a smear negative PTB diagnostic algorithm adopted from the World Health Organization (WHO) (Figure 1) [6].

This study was conducted with the aim of assessing the sensitivity of the current recommended algorithm for the diagnosis of sputum smear negative PTB.

## Methods

A cross-sectional study with prospective enrollment was conducted at Muhimbili National Hospital (MNH), a university teaching and national referral hospital, and at out-patient tuberculosis clinics at the Infectious Disease Clinic (IDC), Mwananyamala, Temeke and Ilala district hospitals, from September 2000 to December 2000. All these hospitals are located in the city of Dar es Salaam. The city accounts for over 26% of all new tuberculosis cases reported each year in Tanzania [7].

Adult male and female patients aged 18 to 75 years, presenting with chronic cough (≥2 weeks); who were three times sputum smear-negative for AFBs (ZN stain); and who gave a written informed consent to participate in the study and for HIV testing were included into the study. Patients with known tuberculosis or who had PTB in the past, on anti-TB for treatment or prophylaxis; those with known chronic respiratory diseases (e.g. bronchial asthma, chronic obstructive pulmonary disease, bronchiectasis), those with misplaed HIV results, contaminated cultue results and those with heart failure were excluded. The study protocol was approved by the MUHAS Ethical Review Committee.

### Study procedures

A detailed medical history and physical examination was done by a study clinician and the findings were recorded on a clinical record form. The investigators did not interfere in the treatment of these patients. The treatment centre followed the diagnostic algorithm for smear negative. Laboratory tests included a complete blood count (coulter counter model, manufacturer, city and country) which included estimation of haemoglobin, red blood cell count and indices; and white blood cell count both total and differential. A peripheral blood smear for assessment of red cell morphology was also made. Erythrocyte sedimentation rate (ESR) was set using the Westergren method within 2 hrs of drawing blood.

HIV testing was done according to the Tanzania National AIDS guidelines. Each patient received pre- and post-test counseling and the HIV test was done using a dual ELISA algorithm. Sera which were non-reactive on first ELISA were considered HIV antibody negative, and those reactive on first ELISA were retested by a second ELISA based on a different test principle. Sera reactive on both ELISA tests were considered HIV-positive. Samples with discordant test results were confirmed by Western blot (WB) and western blot interpretation was done according to the WHO criteria [8].

Patients who came to the clinic with symptoms suggestive of PTB had their sputum examined. Those who were three times smear negative were consequently selected and asked to bring one more sputum sample which was sent to the Tuberculosis Reference Laboratory at MNH for AFB culture (Löwenstein-Jensen culture media).

Smears were considered positive if AFBs were seen on smear from any of the three sputum samples. Patients found to have sputum smear positive were treated for tuberculosis according to the National Tuberculosis and Leprosy program treatment guidelines. Those found to have sputum smear negative for AFB and who consented were enrolled into the study. A chest x-ray was ordered for those who were found to be HIV positive. If the chest x-ray results were abnormal, the patient was considered to have sputum smear negative PTB, and started on anti-TB medications. The rest were treated with broad-spectrum antibiotics. All enrolled patients were requested to stay at the clinic for a month for follow up. Two weeks later, patients on broad spectrum antibiotics were evaluated again by doing sputum smear and chest radiograph. Those found to have smear negative sputum but had symptoms still suggestive of PTB were treated as smear negative PTB; others were treated accordingly.

Two weeks later we came back to review treatment of clinician of which others were given ant TB and others were treated for other respiratory problems. Because they followed NTLP diagnostic algorithm, all of them had a chest X-ray done, and all of them were reviewed and reported using a structured format by two independent radiologists. In case of disagreement in their initial independent reporting, they reviewed the radiographs together and resolved the disagreement by consensus.

### Sample size and data analyisis

#### Power calculations

This study was part of another study on that aimed to investigate/examine sputum smear negative but culture positive PTB the association with HIV.

Assuming a sensitivity of 50% and specificity of 75%, a sample of size 413 subjects would give a 95% confidence interval of plus/minus 0.048% for sensitivity and plus/minus 0.042% for specificity. This is a reasonable amount of precision for the given sample size.

Data were analyzed using Statistical Package for Social Sciences (SPSS) and EPI Info. Pearson chi-square test was used for comparison of categorical data and a student t-test was used for continuous data. Logistic regression analysis was applied and the direct effects of the predictors were assessed by their 95% confidence intervals. A p-value of < 0.05 was considered to be statistically significant.

Sensitivty and specificity of the diagnostic algorithm was calculated using the following formulas:

$$\begin{array}{c} \text{Sensitivity}=\frac{\text{Diseased}}{\text{Positive test}} \end{array}$$

$$\begin{array}{c} \text{Specificity}=\frac{\text{Health individual}}{\text{Test negative}} \end{array}$$

Positive and negative predictive value was calculated using the following formlas

$$\begin{array}{c} \text{Positive predictive value}=\frac{\text{TP}}{\text{TP}+\text{FN}}=\frac{\text{True positive}}{\text{Total positive}} \end{array}$$

$$\begin{array}{c} \text{Negative predictive value}=\frac{\text{TN}}{\text{TN}+\text{ FP}}=\frac{\text{True negative}}{\text{Total negative}} \end{array}$$

## Results

Over the course of the study, 467 patients were enrolled. Of those enrolled, 318 (68.1%) were HIV positive. Sputum was culture positive for MTB in 27.2% (127/467) of patients; samples of 11.1% (52/467) patients were reported to have been contaminated. In the remaining 61.7% (288/467) patients sputum was culture negative for MTB at 8 weeks. Of the 467 study subjects, 68.1% (318/467) were HIV positive. Two study subjects whose culture results were negative had their HIV results misplaced. These two, together with the 52 subjects whose culture results were contaminated were excluded from further analysis. Of the 413 samples analyzed, 30.8% (127/413) were MTB culture positive.

There was a high proportion of PTB patients who were not treated as well as a high proportion of patients without TB who were treated with anti TB [Table 1]. As observed the diagnostic procedure at the clinics had a sensitivity of 38.1% and a specificity of 74.5%. The positive predictive and the negative predictive diagnostic value were 52% and 62.5% respectively [Table 2].

**Table 1:** Diagnosis made using culture results and Treatment given

Treatment given	Culture +ve (n = 127)	Culture -ve (n = 286)	Total (n = 413)
Ant-TB	66(51.9)	107(37.4)	173(41.8)
Antibiotics without anti-TB	61(48.1)	179(62.6)	240(58.2)

**Table 2:** Sensitivity and specificity of the diagnostic procedure of patients with smear negative

**TB treatment**	**Disease status**		**Total**
	
	**Culture +ve**	**Culture -ve**	
Yes	66	107	173
No	61	179	240
Total	127	286	413

Positive predictive value = 52%

Negative predictive value = 62.6%

Sensitivity = 38.1%

Specificity = 74.5%

Of those who were presumptively diagnosed to have TB, the diagnosis of TB was established in HIV negative (58.1%) more than positive subjects (48.8%) [Table 3].

**Table 3:** Diagnosis made and Treatment given by subjects' sputum culture results and HIV sero-status.

**Treatment given**	**Culture +ve**		**Culture -ve**	
	
	**HIV +ve**	**HIV -ve**	**HIV +ve**	**HIV-ve**
	**n = 84**	**n = 43**	**n = 203**	**n = 83**
Anti-TB	41(48.8)	25(58.1)	70(34.5)	37(46.3)
Antibiotics	43(51.2)	18(39.5)	133(65.5)	46(53.7)

Using an unadjusted logistic regression model characteristics which predicts smear negative culture positive were determined. Matted lymph node, tachypnoea (RR > 20), presence of a cavity, mixed type of anaemia, were strong predictors of PTB culture positivity. Eosinophilia was also found to be associated with a 50% less chance of being sputum culture positive [Table 4].

**Table 4:** Unadjusted bivariate logistic regression analysis of clinical characteristics predictive of smear negative culture positive PTB

Characteristics		OR	Confidence interval	P-value
**Resp rate**	> 20/min	2.5	1.3-4.8	**0.005**
	< 20/min	1		
**Lymphnode**	None	1		
	Matted	2.8	1.1-7	**0.03**
	Discrete	1.2	0.5-2.7	0.6
**Eosinophilia**	< 0.4	1		
	> 0.4	0.5	0.2-0.8	**0.01**
**Cavity**	Abscent	1		
	Present	74	9.2-598	**0.0001**
	Cavity right mid	11	0.8-139.7	**0.06**
	Cavity other sites			NS
**RBC morphology**	Normocytic	1		
	Microcytic	0.8	0.4-1.7	0.6
	Macrocytic	0.01	0.000-407516	0.7
	Mixed	2	1.1-3.5	**0.003**

In an attempt to develop supplemental method for diagnosing smear negative pulmonary tuberculosis, forward stepwise multiple logistic regression analysis of the data was done to establish clinical and laboratory characteristics that predict the presence of sputum culture positive. Using this analysis, high respiratory rates, low eosinophil counts, mixed type of anaemia and the presence of cavities on X-rays were predictors of smear negative but culture positive[Table 5].

**Table 5:** Multivariate analysis of clinical characteristics predictive of smear negative culture positive PTB

Characteristics		OR	Confidence interval	P-value
**Respiratory rate**	> 20/min	2.4	1.2-4.4	**0.0054**
	< 20/min	1		
**Eosinophilia**	< 0.4	1		
	> 0.4	0.5	0.2-0.8	**0.0094**
**Cavity**	Abscent	1		
	Present	74.3	9.4-587	**0.0000**
**RBC morphology**	Normocytic	1		
	Microcytic	0.8	0.4-1.7	0.6
	Macrocytic	0.01	0.000-39171193	0.69
	Mixed	2	1.2-3.7	**0.01**

## Discussion

This study showed that of those found to have a negative result for AFB, a significant proportion (27.2%) had sputum culture positive for MTB. Therefore our data indicate that smears did not detect PTB in a very large proportion of patients. Sputum culture being the gold standard for the diagnosis of Tuberculosis disease [9] shows that sputum smear is not a very sensitive tool in the diagnosis of PTB. This has been shown by other studies where sensitivity has been described to be between 51% to 53.3% [10,11]. One of the reasons for low sensitivity is reported to be due to the fact that 10^4^/ml are required for AFB to be seen using smear microscopy [4].

Although the gold standard for the diagnosis of Tuberculosis involves the isolation and identification of Mycobacterium Tuberculosis (MTB) using cultures [9], the cost and facilities of doing cultures are prohibitive in most developing countries. Sputum smear microscopy remains the main diagnostic tool for PTB that allows initiation of treatment and monitoring of patient progress [11,12]. As sputum smear and microscopy is not a very sensitive tool in the diagnosis of PTB, presumptive diagnosis is usually made based on an algorithm of clinical and radiological criteria. This is commonly termed as AFB negative PTB [9,13]. In some cases when sputum smears are negative but the patient has clinical features highly suggestive of PTB, broad-spectrum antibiotics are recommended for at lest 10-14 days and sputum smears repeated. If the patient's condition does not improve while sputum smear remains negative, a chest radiograph is done and if found to be abnormal, a presumptive diagnosis of PTB is made and the patient is started on anti-Tuberculosis treatment as AFB negative PTB [9]. In this study patients whose sputum smears were AFB negative, were evaluated using the above algorithm by the treating doctors at the clinics or hospital. A presumptive diagnosis of AFB sputum smear negative PTB was made in 41.8% (173) of all study subjects, and patients were started on anti-TB treatment as recommended by the Tanzania NTLP. The remaining 58.2% (240) patients were assumed to have other forms of respiratory diseases and were treated accordingly.

Less than half (38.1%) of those who were presumed to have active TB and started on Anti TB actually had TB by sputum culture results. More than 60% of these patients they received 8-months of treatment despite having a negative culture results. This is similar to what has been reported before in Malawi were it was reproted that 40% of smear negative had TB confirmed microbiologically after taking Broncho alveolar larvage [14]. The treatment of individuals without tuberculosis adds to the cost of the TB programs in most developing countries. Likewise about 48% (61) of patients who had active tuberculosis by the results of sputum culture were missed and they received inappropriate treatment, leaving them vulnerable to developing severe disease as well as remaining source of TB infection in the community.

The current diagnostic algorithm leading to the establishment of the diagnosis of AFB smear negative PTB is inefficient; it over-diagnoses PTB and misses a lot of people with active disease. Instituting a more sensitive diagnostic tool will prevent the unnecessary cost of treating individuals who do not have TB and at the same time it will prevent the further spread of TB. This emphasizes the need of culture and the need of further research in order to identify a better diagnostic tool for diagnosis of AFB negative PTB.

In an attempt to improve on the diagnostic algorithm, the study looked at the clinical presentation of the patients to identify clinical laboratory and radiological features that are associated with smer negative PTB and which can be used to predict PTB in patients with symptoms suggestive of PTB. A multivariate analysis showed the following features to be highly predictive of AFB negative but culture positive PTB; low eosinophil counts, a mixed type of anaemia and the presence of cavities on chest radiographs. Low eosinphil seems to be an incidental finding Further studies have to be done to confirm this findings

Limitation in the current study is the inclusion of patients with cough of more than two weeks in which there may be inclusion of patients with simple chest infection that sometime may be complicated with cough for 2-3 weeks. This may be a selection bias that may explain the low sensitivity and specificity of the diagnostic algorithm.

Another limitation is the method of sputum delivery, which is delivered by the patient himself, may have affected the results as some might bring saliva.

We could not be certain that the algorithm was followed at all times because resechers were not involved in the management of these patients rather we evaluated the treatment gien to patients by the attending clinicians. In Tanzania National TB and Leprosy programme is well organized and the algorithm is well adhered by the District TB and Leprosy Coordinators and all workers of the NTLP who were the attending clinicians in this study

## Conclusion

• The current procedures of establishing AFB negative PTB are not sensitive enough to establish the diagnosis of active tuberculosis. They under-diagnose PTB and over treat people without PTB.

• The presence of a dry cough, a high respiratory rate, a low eosinophil count, a mixed type of anaemia and presence of a cavity were found to be predictive of smear negative but culture positive PTB.

## Consent

The study protocol was approved by the MNH Ethical Review Committee.

Written consent was obtained. To those who were not able to write oral consent was obtained

The strength of our study is that it evaluates very well the dignostic algorithm.

### Recommendations

1. We do not recommend adhering to the diagnostic algorithm.

2. A much more sensitive diagnostic algorithm for smear negative pulmonary tuberculosis should be developed to be able to identify those individuals who are actually sputum culture positive for AFB.

## List of Abbreviations

HIV: Human Immunodeficiency Virus; PTB: Pulmonary Tuberculosis; AFB: Acid Fast bacilli; MTB: Mycobacterium Tuberculosis Mycobacterium Tuberculosis; NTLP: National Tuberculosis and Leprosy Proagramme; ZN: Ziehl-Nelseen; IDC: Infectious Disease Clinic; TB: Tuberculosis; Anti-TB: Anti-tuberculosis drugs; RR: Respiratory rate; MNH: Muhimbili National Hospital; WHO: World Health Organization; ELIZA: Enzyme Immunosorbent assay; SPSS: Statistical Package for Social Sciences; OR: Odds ratio.

## Competing interests

The authors declare that they have no competing interests.

## Authors' contributions

FM participated in the design of the study, proof read the manuscript and performed statistical analysis. HS participated in the design of the study, collect data, drafted the manuscript, and performed statistical analysis. JM participated in the statistical analysis and proof read of the manuscript. All authors' read and approved the manuscript.
